# Dietary diversity, frailty transitions, and frailty-state life expectancy in Chinese older adults: a 10-year multistate analysis of the CLHLS

**DOI:** 10.3389/fnut.2026.2006988

**Published:** 2026-11-13

**Authors:** Lin Zhou, Daoze Ke, Simin Liao, Junru Li, Hang Dong

**Affiliations:** 1 The First Clinical College of Guangzhou University of Chinese Medicine, Guangzhou, China; 2 ShunDe Hospital of Guangzhou University of Chinese Medicine, Foshan, China; 3 The First Affiliated Hospital of Guangzhou University of Chinese Medicine, Guangzhou, China

**Keywords:** Chinese longitudinal healthy longevity survey, dietary diversity, frailty, healthy aging, life expectancy, multistate model, oldest-old, prefrailty

## Abstract

**Background:**

Dietary diversity has been linked to incident frailty, but it remains unclear whether it is associated with specific transitions along the frailty continuum and with the number of later-life years spent in healthier states. We examined dietary diversity in relation to frailty transitions and frailty-state life expectancy in Chinese older adults.

**Methods:**

We analyzed 2008–2018 data from the Chinese Longitudinal Healthy Longevity Survey. Frailty was classified as robust, prefrail, or frail using a graded deficit-accumulation frailty index (FI), and dietary diversity was assessed with a repeated 0–18 dietary diversity score (DDS). Multistate models estimated transition intensity ratios (IRs) with 95% confidence intervals (CIs) for transitions among frailty states and death, and a previous-wave analysis separated dietary assessment from subsequent transitions. Frailty-state life expectancy was estimated at ages 65, 70, 75, and 80 years.

**Results:**

The primary analysis included 22,804 person-period observations from 13,427 individuals. In the previous-wave analysis, each 2-point higher DDS was associated with lower robust-to-prefrail transition intensity (IR 0.892, 95% CI 0.835–0.953), whereas the interval-start DDS estimate within the same 9,464 intervals was 0.982 (0.922–1.046). The lagged association was similar under FI threshold and dietary-component sensitivity analyses. Under model-standardized age-65 scenarios, DDS 11 versus DDS 7 corresponded to +0.45 robust years (95% uncertainty interval [UI] + 0.23 to +0.70), whereas the +0.23 non-frail and +0.20 total-year contrasts had UIs including zero. Life-expectancy contrasts depended on the fitted model and standardization assumptions.

**Conclusion:**

Higher DDS recorded at the preceding wave was associated with a lower subsequent intensity of early frailty deterioration. Model-standardized higher-DDS scenarios showed more years in the robust state; at age 65, uncertainty intervals for differences in total and non-frail years included zero. The observational results do not establish that dietary changes prevent frailty or preferentially affect deterioration over recovery.

## Introduction

Frailty is a dynamic process rather than a fixed health state (1–3). Older adults may deteriorate from robust to prefrail or frail states, remain stable, recover to a less severe state, or die (4–6). This dynamic nature is clinically important because a modifiable exposure may influence one part of the frailty continuum more strongly than another. Conventional incident-frailty analyses capture the first occurrence of frailty but provide limited information on whether an exposure is associated primarily with early deterioration, recovery, or the amount of later life spent in healthier versus frailer states.

Nutrition is one of the most modifiable determinants of frailty. Higher-quality dietary patterns, including Mediterranean-style diets, have generally been associated with lower risks of frailty or prefrailty (7–10), while protein intake has also been linked to transitions between frailty states (11–13). These findings suggest that nutritional exposures may act differently across stages of frailty. However, evidence on transition-specific dietary associations remains limited.

Dietary diversity provides a practical indicator of habitual food variety, particularly in older populations in whom repeated quantitative assessment of energy and nutrient intake can be difficult. In the Chinese Longitudinal Healthy Longevity Survey (CLHLS), higher dietary diversity and more favorable changes in dietary diversity have been associated with lower incident frailty and healthier aging outcomes (14–16). Dietary diversity has also been linked to mortality (17–19), cognitive health (20–22), and other health indicators in Chinese older adults (23, 24). What remains unclear is whether greater dietary diversity is preferentially associated with particular transitions along the frailty continuum and whether it corresponds to more remaining years lived in robust or non-frail states.

The CLHLS provides a distinctive setting in which to address these questions because of its repeated longitudinal assessments and unusually strong representation of adults at advanced ages (25). We therefore examined dietary diversity in relation to bidirectional transitions among robust, prefrail, and frail states and to death, and estimated frailty-state life expectancy across different levels of dietary diversity (26). We also used previous-wave dietary diversity to strengthen temporal separation between dietary assessment and subsequent frailty transitions. Our aim was to determine where along the frailty continuum dietary diversity was most clearly associated with change and whether higher dietary diversity corresponded to more remaining years lived in healthier frailty states.

## Materials and methods

### Study design and population

This longitudinal cohort study used the 2008, 2011/12, 2014 and 2018 waves of the Chinese Longitudinal Healthy Longevity Survey (CLHLS), a nationwide survey with substantial representation of adults at advanced ages (25). Observations were organized into three successive transition intervals: 2008–2011/12, 2011/12–2014 and 2014–2018; the final wave was an endpoint rather than the beginning of a fourth transition interval. These are person-period records, and a participant could contribute more than one interval. The reconstructed longitudinal data comprised 24,501 eligible intervals from 13,790 participants; the primary complete-covariate analysis included 22,804 intervals from 13,427 participants (Figure 1). Follow-up for the present analysis ended in 2018.

**Figure 1:**
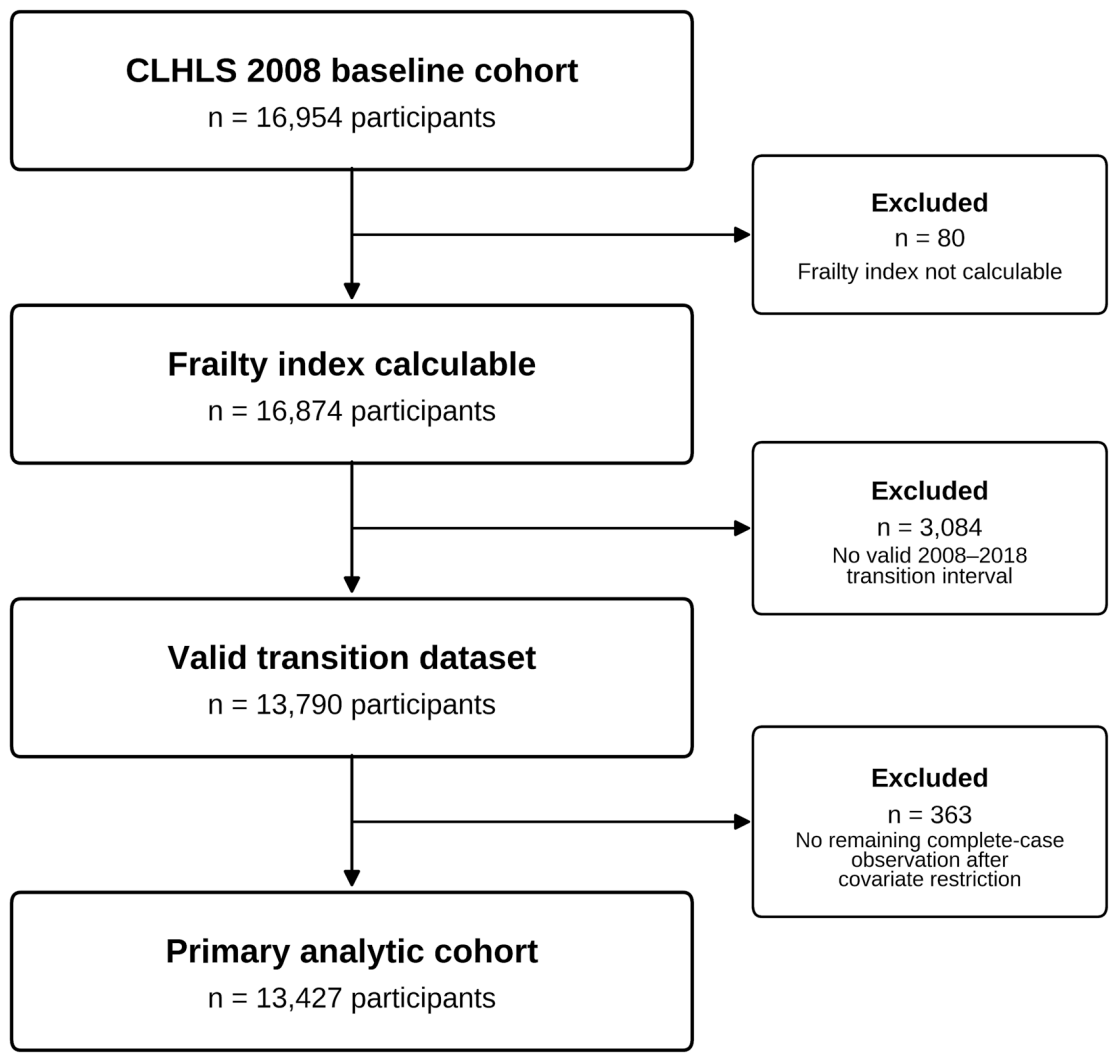
Participant selection for the primary analytic cohort. The primary analysis included 13,427 participants and 22,804 person-period observations; mortality follow-up ended in 2018.

### Frailty and dietary diversity assessment

Frailty was measured using a graded deficit-accumulation frailty index (FI) combining 38 functional, sensory, cognitive, psychological, disease and health-rating components (27–30). Components were scored between 0 and 1 when available; the serious-illness component had a maximum weight of two deficit units, yielding 39 possible deficit units in total. The numerator was the sum of observed deficit scores and the denominator was the sum of available item capacities; if the serious-illness item was missing, two units rather than one were excluded from the denominator. FI was considered unavailable if fewer than 70% of the 39 total units were assessable (fewer than 28 units). The primary thresholds were robust (FI ≤ 0.10), prefrail (0.10 < FI ≤ 0.21), and frail (FI > 0.21), consistent with the earlier CLHLS frailty-state life-expectancy framework (26). Full item codes, unit capacities and executed recoding rules are supplied in Supplementary Table S5.

The primary exposure was a time-varying, frequency-weighted dietary diversity score (DDS) comprising nine food groups: fresh fruit, fresh vegetables, legumes/beans, salted vegetables, garlic, tea, meat, fish and eggs. For the seven five-level frequency items, original response 1 was assigned 2 points, responses 2–4 were assigned 1 point, and response 5 was assigned 0 points; for four-level fresh-fruit and fresh-vegetable items, responses 1, 2–3 and 4 were assigned 2, 1 and 0 points, respectively (Supplementary Table S6). The nine components sum to 0–18 points. DDS was updated at the start of each transition interval and entered the primary multistate model continuously, with intensity ratios reported for each 2-point increment. Low (≤7), middle (8–10) and high (≥11) groups describe the sample distribution; these are not inferential cut-points selected to optimise the observed associations.

### Covariates

Models were adjusted for age, sex, urban residence, farmer occupation, marital status, perceived income sufficiency, current smoking, current drinking, regular exercise, body mass index, number of natural teeth, denture use and literacy. In both interval-start and previous-wave DDS models, the covariates refer to the start of the transition interval (2008, 2011/12 or 2014 as applicable), rather than being uniformly shifted back with the dietary exposure. Potential residual confounding from cognitive status, proxy responding and social support was considered in interpreting the observational associations. Proxy-assisted dietary reports and cognitive impairment may affect exposure ascertainment; cognition is also represented within the frailty-index state definition and was not entered again as an independent adjustment variable. Marital status and perceived income sufficiency capture limited aspects of social circumstances, not direct social-support measures.

### Multistate and frailty-state life-expectancy analyses

We fitted a continuous-time Markov multistate model with four states: robust, prefrail, frail, and death, with death specified as an absorbing state (31–34). The primary model allowed bidirectional transitions between adjacent living states (robust ↔ prefrail ↔ frail) and transitions from each living state to death. Observed non-adjacent changes between robust and frail states were not treated as direct instantaneous transitions in the primary model; instead, the model allowed unobserved passage through the intermediate prefrail state between survey assessments. A sensitivity model additionally allowed direct robust ↔ frail transitions, with model convergence and information criteria used to compare alternative transition structures.

The previous-wave DDS analysis was intended to separate measurement of diet from the subsequent frailty-transition interval. DDS from 2008 was linked to transitions observed during 2011/12–2014, while DDS from 2011/12 was linked to transitions during 2014–2018. All covariates in these models remained measured at the corresponding transition-interval start. Of 9,501 primary-model intervals beginning in 2011/12 or 2014, 9,464 (99.6%) had the required previous-wave DDS and entered the lagged analysis; the first 2008–2011/12 interval was not eligible because no prior dietary assessment was present within this analysis window. To distinguish exposure-timing effects from differences in the analytic sample, we also fitted the interval-start DDS model in these same 9,464 intervals, with the same transition structure and covariates (Supplementary Tables S7–S8).

Frailty-state life expectancy was calculated from the fully adjusted primary seven-edge multistate model. We projected age-specific expected occupation of robust, prefrail and frail states from starting ages 65, 70, 75 and 80 years under fixed DDS values of 7, 9 and 11 (the observed 25th, 50th and 75th percentiles). Attained age increased continuously during prediction until age 120, while each DDS scenario remained fixed and other covariates were set to their means in the main analytical person-period data. The initial frailty-state distribution was estimated by multinomial logistic regression of baseline 2008 observations (n = 16,467) conditional on starting age and DDS. The expected years in each living state were integrated over the prediction period; non-frail life expectancy was defined as robust plus prefrail years. These are model-standardized scenario estimates, not intervention effects.

Model comparison used the Akaike information criterion (AIC) and Bayesian information criterion (BIC). Transition-specific associations are reported as intensity ratios (IRs) with 95% confidence intervals (CIs). For the seven-edge models, uncertainty was quantified from the observed-information covariance matrix. Life-expectancy 95% uncertainty intervals (UIs) were estimated using the 2.5th and 97.5th percentiles of 500 joint draws of the transition-model coefficients; the initial-state model uncertainty was not included. Targeted checks included alternative FI threshold (0.25), a stricter minimum FI information requirement (80% of potential deficit units), an eight-item DDS excluding salted vegetables, and a joint likelihood-ratio test of seven transition-specific DDS quadratic terms against the linear specification. Analyses were implemented in Python using NumPy, SciPy, PyTorch, and statsmodels; additional diagnostics are shown in the Supplementary material. We additionally assessed the influence of FI values immediately below the robust/prefrail boundary by excluding starting FI values of 0.09–0.10 from robust-start intervals in a targeted sensitivity analysis.

## Results

### Cohort characteristics

The 2008 CLHLS data contained 16,954 participant records, of which 16,874 had a calculable FI. Across the three survey intervals, 24,501 valid person-period records were available from 13,790 participants; 22,804 intervals from 13,427 individuals entered the fully adjusted primary model. These comprised 13,303 intervals beginning in 2008, 6,457 beginning in 2011/12 and 3,044 beginning in 2014. The previous-wave DDS analysis included 9,464 of 9,501 primary-model intervals beginning in 2011/12 or 2014 (Figure 1; Supplementary Table S7).

The descriptive analysis of 13,303 participants with a covariate-complete interval beginning in 2008 had a mean age of 86.7 years and a mean DDS of 9.22. Importantly, 3,622 participants were aged 65–79 years at that interval start, including 1,148 aged 65–69; thus model standardization at ages 65–80 is supported by observations in younger-old as well as the oldest-old, although estimates remain model-dependent. At the interval start, 22.8% were robust, 49.7% prefrail and 27.5% frail. Participants with higher DDS were descriptively younger and more often urban residents, married, physically active, literate, financially sufficient and had more natural teeth (Table 1). Wave-specific FI availability is given in Supplementary Table S1 and Supplementary Figure S1.

**Table 1:** Baseline characteristics according to dietary-diversity category.

Characteristic	Overall (N = 13,303)	Dietary-diversity category		
		Low ≤7 (*N* = 3,341)	Middle 8–10 (*N* = 5,777)	High ≥11 (*N* = 4,185)
Age—yr	86.68 ± 11.53	89.51 ± 10.96	86.82 ± 11.37	84.23 ± 11.65
Dietary-diversity score	9.22 ± 2.63	5.85 ± 1.35	9.02 ± 0.81	12.18 ± 1.29
Body-mass index—kg/m^2^	20.28 ± 3.57	19.56 ± 3.42	20.21 ± 3.55	20.95 ± 3.59
No. of natural teeth	7.87 ± 9.47	5.55 ± 8.00	7.48 ± 9.32	10.26 ± 10.21
Sex—no. (%)				
Male	5,767 (43.4)	1,180 (35.3)	2,445 (42.3)	2,142 (51.2)
Female	7,536 (56.6)	2,161 (64.7)	3,332 (57.7)	2,043 (48.8)
Residence—no. (%)				
Urban	4,846 (36.4)	909 (27.2)	2,106 (36.5)	1,831 (43.8)
Rural	8,457 (63.6)	2,432 (72.8)	3,671 (63.5)	2,354 (56.2)
Occupation—no. (%)				
Farmer	9,321 (70.1)	2,662 (79.7)	4,118 (71.3)	2,541 (60.7)
Nonfarmer	3,982 (29.9)	679 (20.3)	1,659 (28.7)	1,644 (39.3)
Marital status—no. (%)				
Currently married	4,235 (31.8)	775 (23.2)	1,790 (31.0)	1,670 (39.9)
Not currently married	9,068 (68.2)	2,566 (76.8)	3,987 (69.0)	2,515 (60.1)
Income sufficiency—no. (%)				
Sufficient	10,209 (76.7)	2,142 (64.1)	4,491 (77.7)	3,576 (85.4)
Insufficient	3,094 (23.3)	1,199 (35.9)	1,286 (22.3)	609 (14.6)
Smoking status—no. (%)				
Current smoker	2,432 (18.3)	472 (14.1)	1,042 (18.0)	918 (21.9)
Not current smoker	10,871 (81.7)	2,869 (85.9)	4,735 (82.0)	3,267 (78.1)
Drinking status—no. (%)				
Current drinker	2,413 (18.1)	434 (13.0)	999 (17.3)	980 (23.4)
Not current drinker	10,890 (81.9)	2,907 (87.0)	4,778 (82.7)	3,205 (76.6)
Regular exercise—no. (%)				
Yes	3,673 (27.6)	651 (19.5)	1,524 (26.4)	1,498 (35.8)
No	9,630 (72.4)	2,690 (80.5)	4,253 (73.6)	2,687 (64.2)
Denture use—no. (%)				
Yes	3,604 (27.1)	738 (22.1)	1,606 (27.8)	1,260 (30.1)
No	9,699 (72.9)	2,603 (77.9)	4,171 (72.2)	2,925 (69.9)
Literacy—no. (%)				
Literate	4,953 (37.2)	862 (25.8)	2,096 (36.3)	1,995 (47.7)
Illiterate	8,350 (62.8)	2,479 (74.2)	3,681 (63.7)	2,190 (52.3)
Frailty state at interval start—no. (%)				
Robust	3,029 (22.8)	387 (11.6)	1,251 (21.7)	1,391 (33.2)
Prefrail	6,616 (49.7)	1,650 (49.4)	2,951 (51.1)	2,015 (48.1)
Frail	3,658 (27.5)	1,304 (39.0)	1,575 (27.3)	779 (18.6)

Data are mean ± SD (standard deviation) or *n* (%); DDS, dietary-diversity score; baseline *n* = 13,303 (complete 2008 interval/covariates).

### Observed frailty transitions and model selection

Among the 24,501 observed intervals, 5,286 began in the robust state, 11,868 in the prefrail state, and 7,347 in the frail state. From robust, 2,324 intervals ended in prefrailty, 578 were observed as frail at the next assessment, and 725 ended in death. From prefrailty, 1,016 ended in robust, 2,598 in frailty, and 3,964 in death. From frailty, 804 ended in prefrailty, 70 were observed as robust, and 4,705 ended in death (Table 2; Figure 2A).

**Table 2:** Observed frailty-state transitions in the pooled longitudinal dataset.

Starting frailty state	Observed destination—no. (%)			
	Robust	Prefrail	Frail	Death
Robust (*n* = 5,286)	1,659 (31.4)	2,324 (44.0)	578 (10.9)^†^	725 (13.7)
Prefrail (*n* = 11,868)	1,016 (8.6)	4,290 (36.1)	2,598 (21.9)	3,964 (33.4)
Frail (*n* = 7,347)	70 (1.0)^†^	804 (10.9)	1,768 (24.1)	4,705 (64.0)

Percentages use the number of intervals beginning in each starting state. ^†^Observed non-adjacent endpoint changes; these were not direct edges in the primary seven-edge model. Total valid intervals: 24,501.

**Figure 2:**
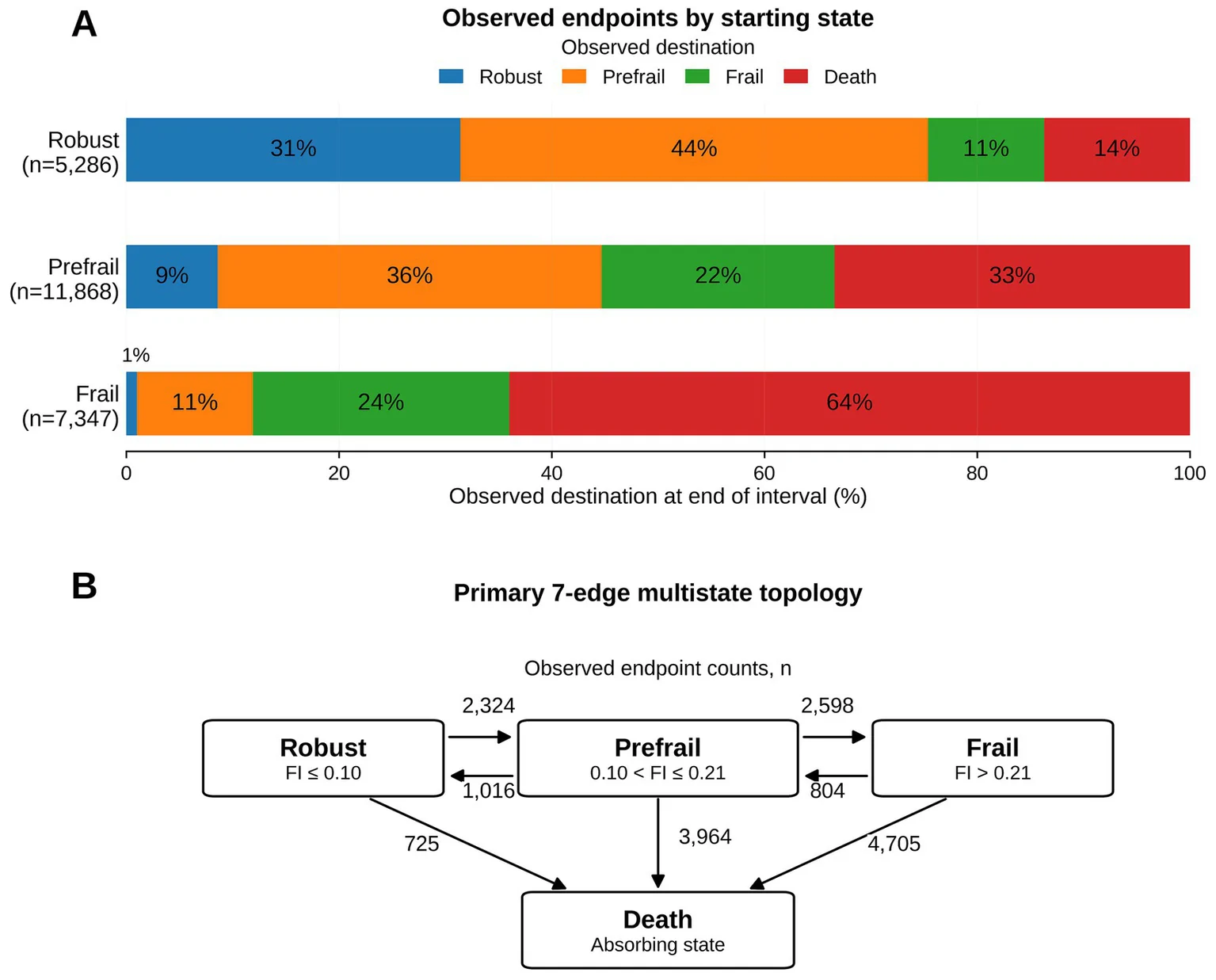
Observed frailty-state endpoints and the primary seven-edge multistate model **(A)** Observed destination states across 24,501 intervals. **(B)** Allowed transitions among robust, prefrail, frail, and death.

The adjacent-state seven-edge model converged and fit better than the nine-edge direct-jump model (AIC 67843.0 vs. 67870.9; BIC 68686.7 vs. 68955.5). The nine-edge model also showed less satisfactory convergence, and the seven-edge topology was retained as the primary model (Figure 2; Supplementary Figure S2; Supplementary Table S3).

### Dietary diversity and frailty-state transitions

Interval-start DDS was not clearly associated with transitions among the three living frailty states. Per 2-point higher DDS, the IR was 1.003 (95% CI 0.959–1.049; *p* = 0.909) for robust to prefrail, 1.053 (0.983–1.127; *p* = 0.141) for prefrail to robust, 1.001 (0.961–1.044; *p* = 0.947) for prefrail to frail, and 1.006 (0.940–1.077; *p* = 0.869) for frail to prefrail (Table 3; Figure 3A).

**Table 3:** Association of dietary diversity with transition intensities in the primary 7-edge multistate model.

Transition	Interval-start DDS 22,804 person-periods		Previous-wave DDS 9,464 person-periods	
	IR (95% CI)	*p*-value	IR (95% CI)	*p*-value
Frailty-state transitions				
Robust → prefrail	1.003 (0.959–1.049)	0.909	0.892 (0.835–0.953)	<0.001
Prefrail → robust	1.053 (0.983–1.127)	0.141	1.014 (0.913–1.126)	0.799
Prefrail → frail	1.001 (0.961–1.044)	0.947	0.987 (0.932–1.045)	0.652
Frail → prefrail	1.006 (0.940–1.077)	0.869	1.056 (0.960–1.162)	0.263
Transitions to death				
Robust → death	0.800 (0.644–0.994)	0.044	1.091 (0.658–1.809)	0.736
Prefrail → death	1.117 (1.040–1.199)	0.002	0.947 (0.804–1.117)	0.520
Frail → death	0.963 (0.938–0.989)	0.005	0.988 (0.949–1.028)	0.540

IR, intensity ratio; CI, confidence interval; DDS, dietary-diversity score. IRs are per 2-point higher DDS. Models were adjusted for the prespecified covariates listed in methods.

**Figure 3:**
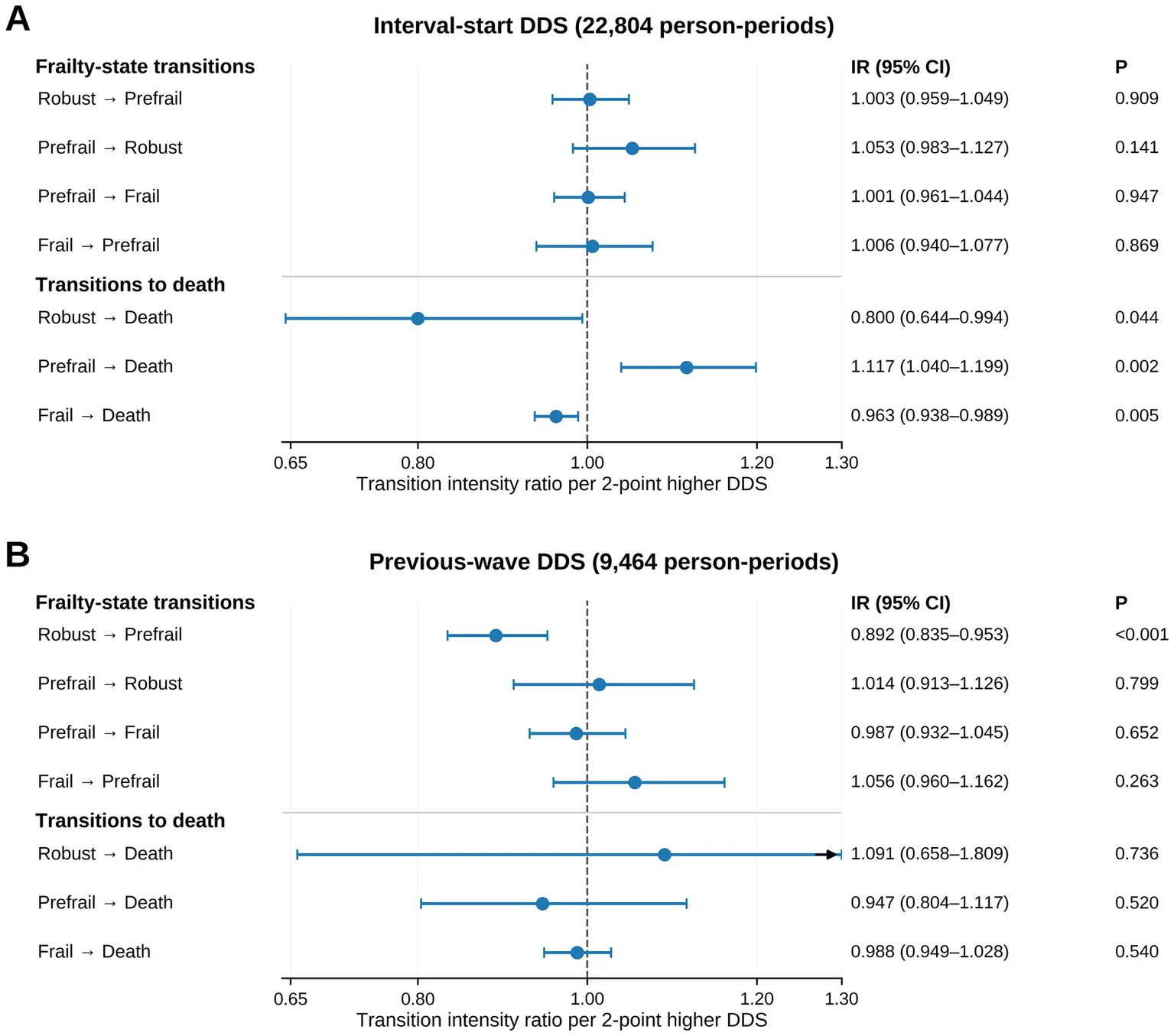
Dietary diversity and frailty-state transitions. Adjusted IRs (95% CIs) per 2-point higher DDS for **(A)** Interval-start DDS and **(B)** Previous-wave DDS.

Interval-start DDS associations with transitions to death differed in direction by starting frailty state: robust to death IR 0.800 (95% CI 0.644–0.994; *p* = 0.044), prefrail to death IR 1.117 (1.040–1.199; *p* = 0.002), and frail to death IR 0.963 (0.938–0.989; *p* = 0.005). This heterogeneous pattern was not replicated when DDS was shifted back one wave and should not be interpreted as evidence of a causal effect on mortality.

In the previous-wave analysis, each 2-point higher DDS was associated with a lower subsequent transition intensity from robust to prefrail (IR 0.892, 95% CI 0.835–0.953; *p* < 0.001). In the identical 9,464 person-period observations, however, the interval-start DDS estimate for this transition was IR 0.982 (95% CI 0.922–1.046; *p* = 0.576). Thus, the larger difference between the full primary and previous-wave estimates cannot be explained solely by losing the 2008–2011/12 intervals; the result is also sensitive to the timing of dietary measurement. Other lagged living-state transition estimates were not clearly associated with DDS, and the interval-start mortality associations were not reproduced in the previous-wave analysis (Figure 3B; Supplementary Table S8). These comparisons do not themselves establish a causal temporal effect.

Sensitivity analyses: The previous-wave DDS association with robust-to-prefrail transition intensity remained similar with an FI frailty threshold of 0.25 (IR 0.891, 95% CI 0.836–0.949), with at least 80% of potential FI deficit units available (IR 0.885, 0.825–0.948), and after removing salted vegetables from the DDS (IR 0.909, 0.846–0.978). A nested comparison that added DDS quadratic terms to all seven transition intensities did not provide strong evidence against the linear specification (LR *χ*^2^ = 9.12, 7 *df*; *p* = 0.244). These checks support the stability of the observed lagged association across the tested definitions but do not eliminate residual confounding or establish a causal effect (Supplementary Table S9). In an additional check addressing near-boundary state changes, we excluded 497 robust-start records with starting FI 0.09–0.10; the previous-wave DDS estimate for the robust-to-prefrail transition remained 0.875 (95% CI 0.812–0.943; 8,967 eligible person-periods).

### Dietary diversity and frailty-state life expectancy

At age 65 and DDS 9, the updated fully adjusted model yielded a model-standardized total remaining life expectancy of 14.95 years (95% UI 14.56–15.33), comprising 3.61 robust years (3.39–3.82), 7.88 prefrail years (7.59–8.18) and 3.46 frail years (3.28–3.60) (Table 4; Figure 4A; Supplementary Table S4). Total remaining life expectancy was 14.82 years at DDS 7 and 15.02 years at DDS 11.

**Table 4:** Model-standardized remaining life expectancy according to starting age and dietary-diversity score.

Starting age/DDS	State-specific remaining life expectancy, yr (95% UI)			Composite remaining life expectancy, yr (95% UI)	
	Robust	Prefrail	Frail	Non-frail	Total
Starting age: 65 yr					
DDS 7 (P25)	3.37 (3.12–3.62)	7.98 (7.64–8.32)	3.47 (3.27–3.63)	11.35 (10.96–11.73)	14.82 (14.39–15.22)
DDS 9 (P50)	3.61 (3.39–3.82)	7.88 (7.59–8.18)	3.46 (3.28–3.60)	11.49 (11.18–11.84)	14.95 (14.56–15.33)
DDS 11 (P75)	3.83 (3.61–4.08)	7.76 (7.44–8.07)	3.44 (3.22–3.60)	11.59 (11.24–11.94)	15.02 (14.56–15.40)
Starting age: 70 yr					
DDS 7 (P25)	2.27 (2.12–2.40)	6.50 (6.27–6.74)	3.26 (3.10–3.39)	8.77 (8.50–9.03)	12.02 (11.70–12.34)
DDS 9 (P50)	2.47 (2.34–2.59)	6.45 (6.25–6.67)	3.24 (3.10–3.35)	8.92 (8.69–9.17)	12.16 (11.87–12.44)
DDS 11 (P75)	2.66 (2.52–2.82)	6.37 (6.14–6.58)	3.22 (3.05–3.34)	9.03 (8.79–9.30)	12.25 (11.91–12.55)
Starting age: 75 yr					
DDS 7 (P25)	1.46 (1.37–1.54)	5.13 (4.96–5.30)	3.03 (2.91–3.13)	6.58 (6.39–6.79)	9.61 (9.36–9.85)
DDS 9 (P50)	1.62 (1.55–1.70)	5.12 (4.98–5.26)	3.01 (2.90–3.10)	6.74 (6.58–6.93)	9.75 (9.55–9.95)
DDS 11 (P75)	1.78 (1.69–1.89)	5.08 (4.93–5.24)	2.98 (2.85–3.08)	6.87 (6.69–7.07)	9.85 (9.60–10.07)
Starting age: 80 yr					
DDS 7 (P25)	0.89 (0.84–0.95)	3.91 (3.77–4.03)	2.78 (2.67–2.87)	4.80 (4.65–4.94)	7.57 (7.38–7.75)
DDS 9 (P50)	1.01 (0.97–1.07)	3.94 (3.84–4.04)	2.76 (2.67–2.84)	4.95 (4.84–5.07)	7.71 (7.56–7.87)
DDS 11 (P75)	1.15 (1.08–1.22)	3.94 (3.83–4.06)	2.73 (2.62–2.82)	5.09 (4.96–5.23)	7.82 (7.65–7.99)

DDS 7, 9, and 11 represent the 25th, 50th, and 75th percentiles. Estimates are model-standardized projections from the refitted, fully adjusted seven-transition model; attained age advances to age 120, DDS is held at each scenario value, and other covariates are fixed at analytical means. The initial state distribution is estimated from 2008 baseline data conditional on age and DDS. UI (uncertainty interval) indicates the 2.5th–97.5th percentile interval from 500 draws of the observed-information parameter covariance; uncertainty in the initial-state model is not included. Non-frail life expectancy is robust plus prefrail; contrasts do not establish causal intervention effects.

**Figure 4:**
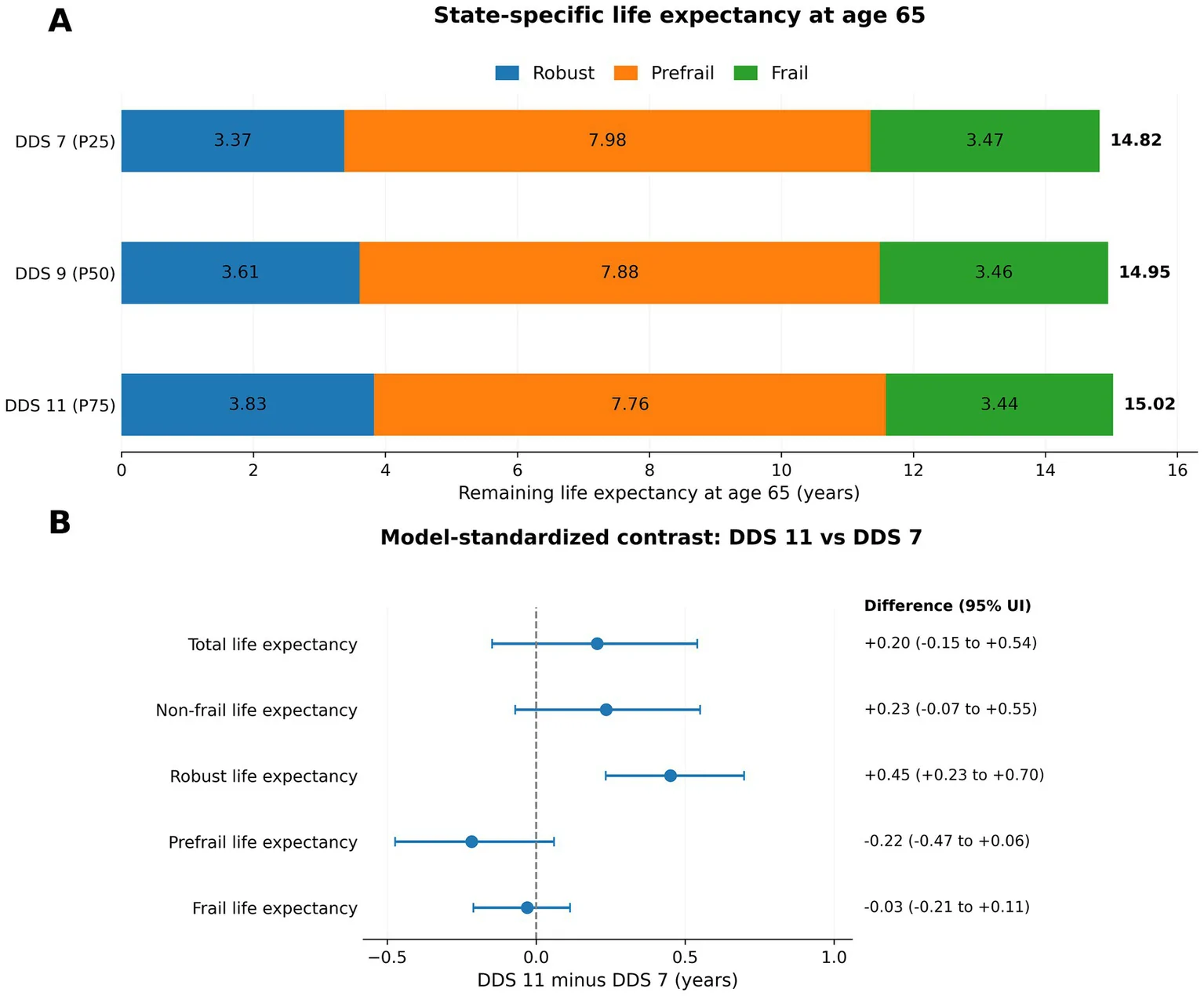
Updated model-standardized frailty-state life expectancy at age 65. **(A)** Remaining expected years in robust, prefrail, and frail states at DDS 7, 9, and 11. **(B)** Model-standardized DDS 11 minus DDS 7 contrast (95% UI from 500 parameter draws). The projections use the fully adjusted seven-edge model with attained age increasing and DDS held fixed.

At age 65, the model-standardized DDS 11 versus DDS 7 contrast corresponded to +0.45 robust years (95% UI + 0.23 to +0.70). The estimated differences were +0.23 non-frail years (−0.07 to +0.55), +0.20 total years (−0.15 to +0.54), −0.22 prefrail years (−0.47 to +0.06), and −0.03 frail years (−0.21 to +0.11). The uncertainty intervals for non-frail and total life expectancy included zero (Figure 4B).

Across starting ages 70, 75 and 80, robust-year differences for DDS 11 versus DDS 7 were approximately +0.39, +0.33 and +0.26 years, respectively; the total-life-expectancy differences were approximately +0.22, +0.24 and +0.25 years, respectively (Figure 5). The precision of these estimates varied with starting age (Supplementary Table S2). Complete state-specific projections are presented in Supplementary Figure S3, and the differences are given in Supplementary Table S2.

**Figure 5:**
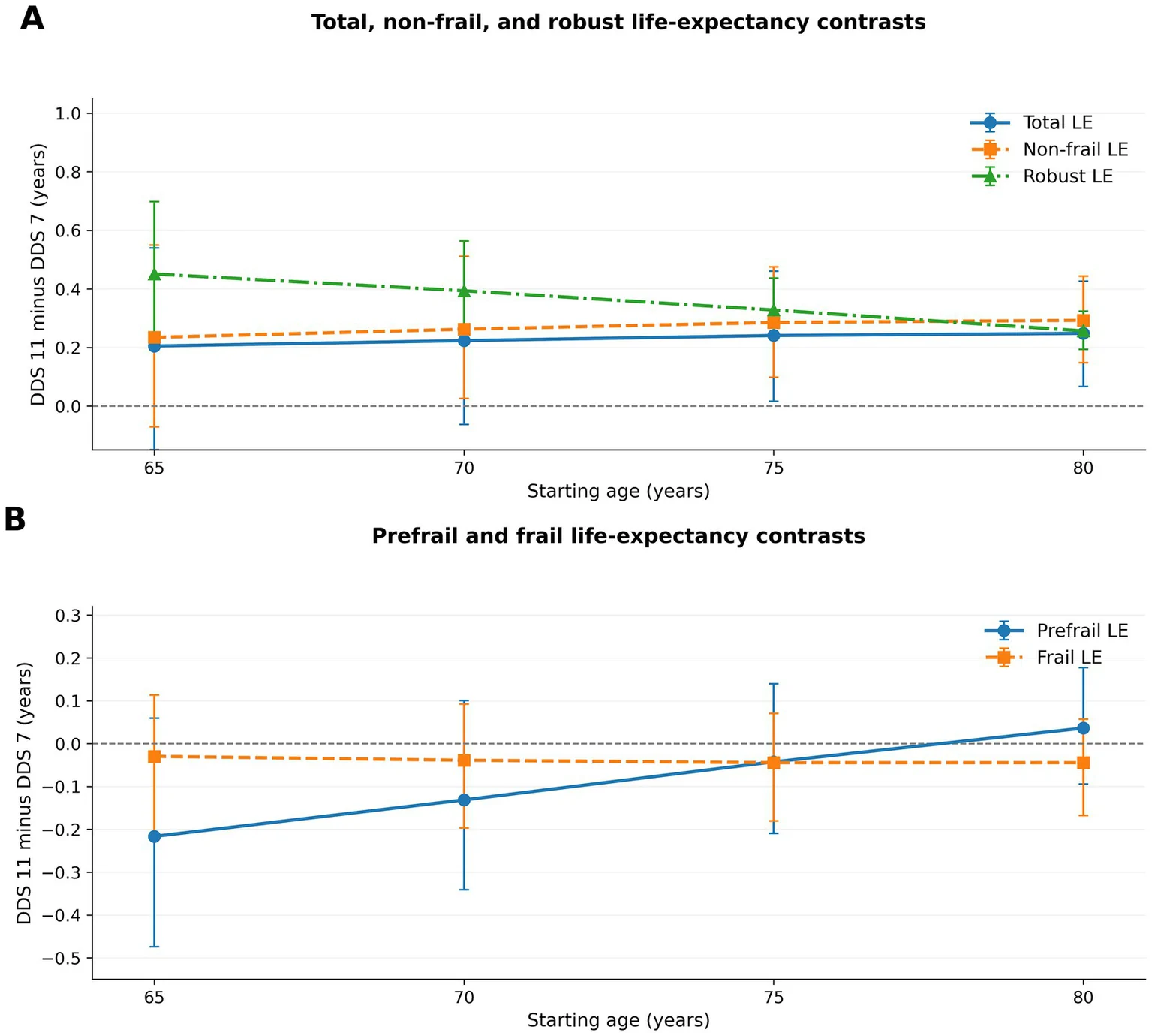
Updated model-standardized differences between DDS 11 and DDS 7 across starting ages. **(A)** Total, non-frail and robust life expectancy. **(B)** Prefrail and frail life expectancy. Error bars indicate 95% parameter-draw uncertainty intervals conditional on the initial-state model.

## Discussion

In this Chinese longitudinal cohort, higher previous-wave dietary diversity was associated with a lower subsequent intensity of transition from robustness to prefrailty, whereas recovery pathways showed no clear association. The updated model-standardized life-expectancy calculations showed higher expected robust-state occupancy under the higher-DDS scenario; at age 65, the contrasts for total and non-frail life expectancy were less precise. These are complementary observational and model-based descriptions, not evidence of a dietary intervention effect.

Previous CLHLS studies have linked greater dietary diversity with lower incident frailty and more favorable frailty trajectories (14–16, 35), but these approaches largely treat frailty as a single outcome or summarize its overall course. Evidence from other longitudinal cohorts also suggests that nutritional exposures may be associated with selected frailty transitions rather than with every possible change (12, 13). By combining transition-specific analysis with frailty-state life expectancy, our study extends earlier CLHLS evidence on dietary diversity and frailty within a single framework (14, 26). The most consistent temporally separated association occurred at the earliest modeled deterioration, from robust to prefrail, whereas recovery transitions showed no clear associations.

Several mechanisms may underlie this pattern. Greater food variety may increase the likelihood of adequate protein, micronutrient, and other dietary intake relevant to muscle function, energy balance, inflammation, and physiologic resilience (7, 11, 36–38). In very old adults, dietary diversity may also reflect the ability to maintain appetite, dentition, mobility, and daily routines sufficient to sustain a broader diet. This context is particularly relevant in the present cohort, in which the mean baseline age was 86.7 years. The association observed with previous-wave DDS is consistent with the possibility that dietary diversity measured before overt deterioration captures longer-term nutritional resilience more effectively than a dietary snapshot taken when health is already changing.

Frailty-state life expectancy illustrates the joint implications of the fully adjusted transition intensities and baseline state distribution under explicitly defined DDS scenarios. At age 65, DDS 11 versus DDS 7 corresponded to approximately 0.45 more robust years in the re-estimated model; non-frail and total life-expectancy differences were smaller and had uncertainty intervals including zero. The estimated contrasts cannot be attributed solely to the previous-wave robust-to-prefrail association because they derive from the interval-start DDS model and combine all transition intensities with the initial-state distribution. Model standardization, age extrapolation, and fixed-DDS assumptions should therefore be considered when interpreting these results (26, 39).

The temporally separated DDS analysis showed an association with robust-to-prefrail transition intensity, whereas the estimated recovery pathways did not show clear associations. We do not infer that an effect on deterioration is statistically stronger than an effect on recovery merely because their individual *p*-values differ. Nutritional interventions have been evaluated in frail and prefrail adults (40), but the present observational findings cannot establish that increasing dietary variety prevents frailty or prolongs healthy life. Further longitudinal and intervention research is needed to test dietary diversity specifically.

Strengths include repeated dietary and frailty assessments, the sizeable national cohort, and complementary transition-specific and state-occupation modeling. Limitations include observational confounding, possible reverse causation, selection into later waves and the lagged exposure window, and a frequency-based score that does not measure energy or nutrient amounts. Frailty states were observed only at discrete survey visits; the FI cut-offs can misclassify small changes near boundaries, although alternative-threshold and completeness checks supported the main lagged association. The heterogeneous interval-start death associations were not retained when DDS was lagged. Life-expectancy projections also rely on assumptions about the initial state distribution, age evolution and holding DDS and other covariates fixed; their reported intervals do not propagate uncertainty in the initial-state model. These modeling assumptions limit causal interpretation. Potential differences between proxy- and self-reported dietary information, cognitive status, and incompletely measured social support may therefore contribute to residual measurement error or confounding.

## Conclusion

Among Chinese older adults, a higher DDS at the preceding survey wave was associated with a lower subsequent intensity of progression from robustness to prefrailty, a pattern that persisted in targeted FI and DDS sensitivity analyses. Under explicit seven-edge model standardization, higher DDS scenarios corresponded to more expected years in the robust state, whereas non-frail and total life-expectancy contrasts were smaller and varied across starting ages. We found no clear evidence of associations with recovery transitions, and these observations cannot establish the causal benefits of changing dietary variety or prove that the exposure acts differently across transition paths.

## Data Availability

The original contributions presented in the study are included in the article/Supplementary material, further inquiries can be directed to the corresponding author.
