# Supplementary material for: Dietary diversity, frailty transitions, and frailty-state life expectancy in Chinese older adults: a 10-year multistate analysis of the CLHLS
Source: Front Nutr. 2026 Nov 13;13:2006988. doi: 10.3389/fnut.2026.2006988 (PMC13760682; doi:10.3389/fnut.2026.2006988)
Supplement: Supplementary file 1 [file Supplementary_file_1.DOCX]

**Supplementary Material**

**Supplementary Figure S1. Frailty distribution and mean frailty index across Chinese Longitudinal Healthy Longevity Survey (CLHLS) waves.** (A) Frailty-state distribution among observations with a valid frailty index. (B) Mean frailty index by survey wave; labels show the number of valid observations. The 2018 wave represents a selected survivor subset.

**Supplementary Figure S2. Sensitivity analysis of multistate topology and model fit.** (A) Primary adjacent-state 7-edge topology. (B) 9-edge sensitivity topology allowing direct Robust↔Frail transitions. (C) Akaike information criterion (AIC) and Bayesian information criterion (BIC) differences for the 9-edge versus 7-edge model; positive values favor the 7-edge model.

**Supplementary Figure S3. Updated model-standardized frailty-state life expectancy by starting age and dietary diversity score (DDS). Predictions begin at ages 65, 70, 75, and 80, with DDS fixed at 7, 9, or 11 and attained age increasing through projection. Error bars show 95% parameter-draw uncertainty intervals conditional on the 2008 initial-state model; the results do not identify a dietary intervention effect.**

**Supplementary Table S1. Frailty-Index Availability and State Distribution by Survey Wave**

| **Wave** | **Valid FI, no.** | **FI, mean (SD)** | **Frailty state, no. (%)** | | |
| --- | --- | --- | --- | --- | --- |
|  |  |  | **Robust** | **Prefrail** | **Frail** |
| 2008 | 16,874 | 0.184 (0.113) | 3,746 (22.2) | 8,092 (48.0) | 5,036 (29.8) |
| 2011 | 8,209 | 0.190 (0.116) | 1,696 (20.7) | 3,898 (47.5) | 2,615 (31.9) |
| 2014 | 4,951 | 0.188 (0.117) | 990 (20.0) | 2,448 (49.4) | 1,513 (30.6) |
| 2018 | 2,185 | 0.217 (0.101) | 80 (3.7) | 1,175 (53.8) | 930 (42.6) |

FI denotes frailty index. The 2018 observations represent a selected survivor subset and should not be interpreted as an unselected population trend.

**Supplementary Table S2. Difference in Model-Standardized Life Expectancy for DDS 11 Versus DDS 7**

| **Starting age** | **Total LE** | **Non-frail LE** | **Robust LE** | **Prefrail LE** | **Frail LE** |
| --- | --- | --- | --- | --- | --- |
| 65 | +0.20 (−0.15 to +0.54) | +0.23 (−0.07 to +0.55) | +0.45 (+0.23 to +0.70) | −0.22 (−0.47 to +0.06) | −0.03 (−0.21 to +0.11) |
| 70 | +0.22 (−0.06 to +0.51) | +0.26 (+0.03 to +0.51) | +0.39 (+0.24 to +0.56) | −0.13 (−0.34 to +0.10) | −0.04 (−0.20 to +0.09) |
| 75 | +0.24 (+0.02 to +0.46) | +0.29 (+0.10 to +0.48) | +0.33 (+0.23 to +0.44) | −0.04 (−0.21 to +0.14) | −0.04 (−0.18 to +0.07) |
| 80 | +0.25 (+0.07 to +0.43) | +0.29 (+0.15 to +0.44) | +0.26 (+0.19 to +0.32) | +0.04 (−0.09 to +0.18) | −0.04 (−0.17 to +0.06) |

Values are contrasts in model-standardized remaining life expectancy (years), DDS 11 minus DDS 7. 95% UIs are percentiles of 500 joint parameter draws from the newly re-estimated fully adjusted seven-edge model; initial-state-model parameter uncertainty is not included. Comparisons are scenario contrasts, not causal dietary effects.

**Supplementary Table S3. Comparison of the Primary 7-Edge and Direct-Jump 9-Edge Multistate Topologies**

| **Model** | **Person-periods** | **Parameters** | **−Log likelihood** | **AIC** | **BIC** | **Convergence** |
| --- | --- | --- | --- | --- | --- | --- |
| Adjacent 7-edge | 22,804 | 105 | 33,816.52 | 67,843.04 | 68,686.68 | Converged |
| 9-edge incl. direct R↔F | 22,804 | 135 | 33,800.43 | 67,870.86 | 68,955.54 | Not fully converged |

The 9-edge model achieved a slightly lower negative log likelihood but used 30 additional parameters, had higher Akaike information criterion (AIC) and Bayesian information criterion (BIC) values, and did not fully converge. The adjacent-state 7-edge topology was therefore retained as primary.

**Supplementary Table S4. Analysis Audit, Numerical Diagnostics, and Life-Expectancy Calibration**

| **Audit item** | **Result** |
| --- | --- |
| **Analytic sample** | |
| Raw 2008–2018 baseline records | 16,954 participants |
| Valid transition dataset | 24,501 person-period observations from 13,790 individuals |
| Primary complete-case 7-edge continuous-time Markov chain (CTMC) | 22,804 person-period observations from 13,427 individuals |
| Previous-wave DDS analysis | 9,464 complete person-period observations |
| **Numerical diagnostics** | |
| Primary 7-edge convergence | Converged; maximum gradient ≈0.0059 |
| Previous-wave 7-edge convergence | Numerically stable; maximum gradient ≈0.0013 after polishing |
| Primary observed-information Hessian | Positive definite; minimum eigenvalue ≈2.69 |
| Previous-wave observed-information Hessian | Positive definite; minimum eigenvalue ≈0.368 |
| Life-expectancy uncertainty propagation | 500 transition-coefficient draws, all successful; conditional on initial-state model |
| **Life-expectancy calibration** | |
| Age-65 DDS=9 total LE | 14.95 years (95% UI 14.56–15.33) |
| Age-65 DDS=9 robust/prefrail/frail LE | 3.61 / 7.88 / 3.46 years (robust / prefrail / frail) |
| Published CLHLS benchmark at age 65 | Total 14.74; Robust 4.18; Prefrail 7.46; Frail 3.10 years (Gao et al., BMC Medicine 2023) |

Rows describing topology fit and transition parameters refer to the independently reproduced seven-edge transition model. Life-expectancy calibration rows use the updated state-occupation calculation with attained age increasing to age 120, DDS held at 7/9/11, other covariates fixed at analytical means, and baseline-state probabilities estimated from 2008 data. The benchmark is observational external context, not direct calibration to the new projection.

## **Supplementary Table S5. Graded frailty-index items, capacity weights and coding**

This table documents the executed historical grading rules (38 components; maximum 39 deficit units). Raw codes outside the specified mappings were treated as unavailable unless specifically noted.

| **Component** | **CLHLS source code** | **Max units** | **Historical recoding** |
| --- | --- | --- | --- |
| Basic activities of daily living | e1–e6 | 6 | 1→0; 2→0.5; 3→1 |
| Limb/mobility tests | g81, g82, g83 | 3 | 1/2→0.5; 3→0; 4→1 |
| Standing ability | g9 | 1 | 1→0; 2→0.5; 3→1 |
| Book handling | g11 | 1 | 1→0; 2→0.5; 3→1 |
| Chopstick use | g3 | 1 | 1→0; 2→1 |
| Turning 360° | g12 | 1 | 0–4→0; 5–87→0.5; 88→1 |
| Vision | g1 | 1 | 1→0; 2→0.33; 3→0.67; 4→1 |
| Hearing | h1 | 1 | 1→0; 2→0.33; 3→0.67; 4→1 |
| Cognitive impairment | 23 Mini-Mental State Examination (MMSE)-type items + c16 | 1 | Summary 24–30→0; 18–23→0.33; 10–17→0.67; 0–9→1; complete component items required |
| Self-rated health | b12 | 1 | 1–5→0/0.25/0.50/0.75/1 |
| Psychological items (negative-coded) | b23, b26 | 2 | 1–5→1/0.75/0.50/0.25/0 each |
| Psychological items (positive-coded) | b21, b22, b25 | 3 | 1–5→0/0.25/0.50/0.75/1 each |
| Heart-rate/related indicator | g71/g72 (2008), g6 (2011/14), g20 (2018) | 1 | 2008 valid pulse outside 60–100/min→1; 2011+ recorded 1→0, 2→1 |
| Housework | d11a | 1 | 1→0; 2–4→0.5; 5→1 |
| Interviewer-rated condition | h3 | 1 | 1→0; 2→0.33; 3→0.67; 4→1 |
| Serious illness (two-unit component) | g131 | 2 | 0→0; 1→1; 2–87→2; original code 88→2 |
| Natural teeth | g21 | 1 | 0–5→1; 6–11→0.8; 12–17→0.6; 18–22→0.4; 23–27→0.2; 28–32→0 |
| Eleven chronic diseases | g15a1, g15b1, g15c1, g15d1, g15e1, g15f1, g15g1, g15i1, g15k1, g15l1, g15m1 | 11 | Each: 1→1; 2→0 |

FI = sum of observed deficit scores / sum of available deficit-unit capacities. The single serious-illness component has maximum 2 units; if unavailable, it removes 2 from the denominator. The total capacity is 39 if all 38 components are observed. FI was set missing if fewer than 70% of the 39 units were observed (i.e., fewer than 28 available units). For repeated waves, the appropriate suffix (_11, _14, _18) is applied except for expressly year-specific variables.

## **Supplementary Table S6. Frequency-to-score mapping of the nine dietary items**

| **Food group** | **Source variable (at interval start)** | **Scored responses (points)** |
| --- | --- | --- |
| Fresh fruit | d31 | 1→2; 2–3→1; 4→0 |
| Fresh vegetables | d32 | 1→2; 2–3→1; 4→0 |
| Legumes/beans | d4bean2 | 1→2; 2–4→1; 5→0 |
| Preserved/salted vegetables | d4veg2 | 1→2; 2–4→1; 5→0 |
| Garlic | d4garl2 | 1→2; 2–4→1; 5→0 |
| Tea | d4tea2 | 1→2; 2–4→1; 5→0 |
| Meat | d4meat2 | 1→2; 2–4→1; 5→0 |
| Fish | d4fish2 | 1→2; 2–4→1; 5→0 |
| Eggs | d4egg2 | 1→2; 2–4→1; 5→0 |

For repeated assessments the corresponding wave suffix was used. Nine items contribute at most 18 points. The continuous score was the primary multistate exposure, reported per 2-point increment. The low (≤7), middle (8–10) and high (≥11) groups were used for descriptive and model-standardized illustration, not as optimized inferential change-points. The nine food-group scores were added to form the 0–18 continuous DDS and were updated at the corresponding survey wave.

## **Supplementary Table S7. Accounting for analytic intervals and the previous-wave DDS comparison**

| **Population/interval definition** | **Count** | **Interpretation** |
| --- | --- | --- |
| Original 2008 respondent records | 16,954 | Persons (not intervals) |
| Valid five-domain FI in 2008 | 16,874 | Baseline respondents with FI |
| All valid transition intervals | 24,501 | 13,790 participants |
| Fully adjusted primary interval-start analysis | 22,804 | 13,427 participants |
| Primary intervals starting 2008 | 13,303 | 2008→2011/12 |
| Primary intervals starting 2011 | 6,457 | 2011/12→2014 |
| Primary intervals starting 2014 | 3,044 | 2014→2018 |
| Primary later intervals potentially eligible for prior DDS | 9,501 | 6,457 + 3,044 |
| Previous-wave DDS complete intervals | 9,464 | 6,451 + 3,013; 6,657 distinct participants |
| Additional later intervals not retained in lag analysis | 37 | 6 from 2011 start, 31 from 2014 start |

The earliest 2008–2011/12 transition intervals have no prior-wave DDS within the study window; they are excluded by design, not due to attrition from a comparable previous-exposure window. Among 9,501 primary-model intervals beginning in 2011/12 or 2014, 9,464 (99.61%) entered the prior-wave analysis. Participants can contribute more than one interval; counts should not be interpreted as unique persons unless indicated.

## **Supplementary Table S8. Equal-sample comparison of interval-start and previous-wave DDS associations**

The same 9,464 person-period records (6,657 individuals) from 2011/12–2014 and 2014–2018 were used in both fully adjusted seven-edge models. DDS was measured either at the transition-interval start or in the preceding wave; the other covariates remained measured at the transition-interval start. IRs correspond to each 2-point increase in DDS. Confidence intervals are based on the full observed-information Hessian.

| Transition | Interval-start DDS: IR (95% CI); P | Previous-wave DDS: IR (95% CI); P |
| --- | --- | --- |
| R → P | 0.982 (0.922–1.046); 0.576 | 0.892 (0.835–0.953); <0.001 |
| P → R | 0.966 (0.866–1.076); 0.526 | 1.014 (0.913–1.126); 0.799 |
| P → F | 1.019 (0.960–1.081); 0.534 | 0.987 (0.932–1.045); 0.652 |
| F → P | 1.025 (0.925–1.135); 0.643 | 1.056 (0.960–1.162); 0.263 |
| R → D | 0.654 (0.440–0.972); 0.036 | 1.091 (0.658–1.809); 0.735 |
| P → D | 0.983 (0.838–1.153); 0.830 | 0.947 (0.804–1.117); 0.519 |
| F → D | 0.989 (0.949–1.030); 0.596 | 0.988 (0.949–1.028); 0.540 |

Abbreviations: R, robust; P, prefrail; F, frail; D, death; IR, transition-intensity ratio; CI, confidence interval. Differences in within-model statistical significance do not constitute a formal test of the difference between exposure-timing coefficients. These are newly re-estimated sensitivity models on the identical set of intervals.

**Supplementary Table S9. Sensitivity of seven transition-intensity estimates to FI and DDS specifications**

| **Transition** | **Prior-wave DDS FI >0.21** | **FI >0.25** | **FI ≥80% information** | **8-food DDS (no salted vegetables)** |
| --- | --- | --- | --- | --- |
| R → P | 0.892 (0.835–0.953) | 0.891 (0.836–0.949) | 0.885 (0.825–0.948) | 0.909 (0.846–0.978) |
| P → R | 1.014 (0.913–1.126) | 1.012 (0.914–1.121) | 1.014 (0.908–1.131) | 1.040 (0.926–1.167) |
| P → F | 0.987 (0.932–1.045) | 0.968 (0.901–1.040) | 0.964 (0.906–1.027) | 0.971 (0.912–1.035) |
| F → P | 1.056 (0.960–1.162) | 1.097 (0.977–1.230) | 1.021 (0.922–1.131) | 1.067 (0.960–1.186) |
| R → D | 1.091 (0.658–1.809) | 1.124 (0.668–1.890) | 1.102 (0.668–1.818) | 1.155 (0.642–2.077) |
| P → D | 0.947 (0.804–1.117) | 1.005 (0.900–1.122) | 1.003 (0.856–1.177) | 0.956 (0.797–1.147) |
| F → D | 0.988 (0.949–1.028) | 0.981 (0.933–1.032) | 0.970 (0.930–1.013) | 0.982 (0.940–1.026) |

IR (95% CI) per 2-point higher DDS from separate fully adjusted seven-edge models. FI >0.25 and ≥80% FI completeness used the reclassified states and corresponding eligible records. The 8-food score excludes salted vegetables and has a maximum of 16 points rather than 18. Confidence intervals are from observed-information covariance. These comparisons do not test differences between coefficients. The nested seven-term quadratic DDS model did not improve fit materially (LR χ²(7)=9.121; *p* = 0.244); this does not exclude every possible nonlinear form. Near-boundary check: excluding 497 robust-start intervals with starting FI 0.09–0.10 left 8,967 person-periods (6,496 individuals); the previous-wave DDS robust-to-prefrail IR was 0.875 (95% CI 0.812–0.943; *p* < 0.001). This exclusion targets records nearest the robust/prefrail boundary and does not establish absence of other measurement error.
